# Effects of seed mixture sowing with transgenic *Bt* rice and its parental line on the population dynamics of target stemborers and leafrollers, and non‐target planthoppers

**DOI:** 10.1111/1744-7917.12571

**Published:** 2018-04-10

**Authors:** Zhuo Li, Li‐Kun Li, Bin Liu, Long Wang, Megha N. Parajulee, Fa‐Jun Chen

**Affiliations:** ^1^ Department of Entomology Nanjing Agricultural University Nanjing China; ^2^ Texas A&M University AgriLife Research and Extension Center Lubbock Texas USA

**Keywords:** cultivar diversity, occurrence and damage, seed mixture sowing, target and non‐target pests, transgenic *Bt* rice, yield

## Abstract

The widespread planting of insect‐resistant crops has caused a dramatic shift in agricultural landscapes, thus raising concerns about the potential impacts on both target and non‐target pests. In this study, we examined the potential effects of intra‐specific seed mixture sowing with transgenic *Bt* rice (Bt) and its parental non‐transgenic line (Nt) (100% *Bt* rice [Bt_100_], 5% Nt+95% *Bt* [Nt_05_Bt_95_], 10% Nt+90% *Bt* [Nt_10_Bt_90_], 20% Nt+80% *Bt* [Nt_20_Bt_80_], 40% Nt+60% *Bt* [Nt_40_Bt_60_] and 100% Nt rice [Nt_100_]) on target and non‐target pests in a 2‐year field trial in southern China. The occurrence of target pests, *Sesamia inferens*, *Chilo suppressalis* and *Cnaphalocrocis medinalis*, decreased with the increased ratio of *Bt* rice, and the mixture ratios with more than 90% *Bt* rice (Bt_100_ and Nt_05_Bt_95_) significantly increased the pest suppression efficiency, with the lowest occurrences of non‐target planthoppers, *Nilaparvata lugens* and *Sogatella furcifera* in Nt_100_ and Nt_05_Bt_95_. Furthermore, there were no significant differences in 1000‐grain dry weight and grain dry weight per 100 plants between Bt_100_ and Nt_05_Bt_95_. Seed mixture sowing of *Bt* rice with ≤10% (especially 5%) of its parent line was sufficient to overcome potential compliance issues that exist with the use of block or structured refuge to provide most effective control of both target and non‐target pests without compromising the grain yield. It is also expected that the strategy of seed mixture sowing with transgenic *Bt* rice and the non‐transgenic parental line would provide rice yield stability while decreasing the insecticide use frequency in rice production.

## Introduction

Transgenic *Bacillus thuringiensis* (*Bt*) rice (i.e., *Bt* rice) expressing *Cry* toxins have demonstrated excellent control of stemborers, *Sesamia inferens*, *Chilo suppressalis* and *Scirpophaga incertulas*, and leafroller *Cnaphalocrocis medinalis* in laboratory and field trials (Cheng *et al*., 1998; Shu *et al*., 2000; Ye *et al*., 2001; Zhao *et al*., 2004; Ho *et al*., 2006). However, several researchers have investigated that the use of *Bt* rice has lowest control on *S. inferens* (Gao *et al*., 2006). It suggests that the survival of *S. inferens* larvae have genetic resistance to *Bt* rice and may cause the occurrence of this target pest to disadvantage of widespread planting of *Bt* rice. Therefore, it is greatly necessary to consider approaches to develop and deploy *Bt* rice cultivars that would delay the evolution of stemborer resistance before *Bt* rice is put into commercial use. The use of seed mixture sowing with susceptible crop seeds provides a block or structured refuge in which susceptible target pests can survive in transgenic crops (Huang *et al*., 2011; Onstad *et al*., 2011). Some researchers have observed that block refuges for *Bt* corn successfully delayed the evolution of target pest resistance on an area‐wide basis in the USA (Tabashnik *et al*., 2009; Andow *et al*., 2010; Hutchison *et al*., 2010; Huang *et al*., 2011; Kang *et al*., 2012; Hutchison *et al*., 2015). Thus, intra‐specific seed mixture sowing became a common strategy to promote insect resistance management (IRM) for *Bt* corn. Simultaneously, the increased vegetation diversity has been regarded as an ecological approach to suppress insect pests in cotton (Bastola *et al*., 2016). A high biodiversity level could effectively protect crops from diseases and insect pests in the agro‐ecosystem, and it is also beneficial for crops to increase the output per unit area and decrease the use of pesticides and fertilizers (Wolfe, 2000; Zhu *et al*., 2000). A long‐term low biodiversity with monoculture cropping systems would break the ecological balance and thus increase heavy occurrences of insect pests and diseases in the agro‐ecosystem (Guo *et al*., 2007). In recognition of this fact, many researchers have investigated the role of agro‐ecosystem biodiversity in enhancing ecological pest suppression. Wang *et al*. (2007) observed that intercropping rice with *Zizania caduciflora* L. and other wetland crops could effectively suppress the occurrence and spread of insect pests and diseases. Cai *et al*. (2005) found no significant differences in diversity, evenness, and dominant concentration of arthropod communities between the mix‐rows and block cropping patterns, but the species richness and individual density of arthropods in the mixed cropping pattern were significantly lower than those in the block cropping pattern. Moreover, reasonable mixtures of different crop cultivars can avoid the disadvantages triggered by a low crop biodiversity and monoculture system (Van & Harfington, 2007). The strip‐cropping of alfalfa could improve the biological control of wheat aphid, *Macrosiphum avenae* (Ma *et al*., 2007) and of cotton aphid, *Aphis gossypii* (Parajulee *et al*., 1997). The use of mixtures of different rice cultivars could effectively control rice blast disease (Zhu *et al*., 2000). Furthermore, the use of habitat diversity combined with resistant rice cultivars has been regarded as an effective approach in rice pest suppression (Skovgard & Pats, 1996; Landis *et al*., 2000; Smith & McSorley, 2000). Thus, the use of reasonable intra‐specific seed mixture to increase crop genetic diversity is of great significance for optimizing the agro‐ecological environment (Lu, 2003).

Rice, *Oryza sativa* L., is one of the most important crops worldwide, the primary staple food for nearly 3 billion people (FAO, 2004). In China, the agricultural insect pests occurred in 15 million hectares, about half the acreage of which was planted with rice in 2002, resulting in significant insect‐induced economic loss in rice production (Sheng *et al*., 2003). In a rice ecosystem, stemborers, leafrollers and planthoppers are the major groups of insect pests to cause economic loss (Arbab, 2014). Four major Lepidoptera pests of rice are the striped stemborer *C. suppressalis*, the pink stemborer *S. inferens*, the yellow stemborer *S. incertulas*, and the leafroller *C. medinalis*. Recent surveys showed that these Lepidoptera pests caused severe damage in Guangxi Zhuang Autonomous Region (Tian, 2015). In China, with increased crop intensification and improvement in crop production technology, *S. inferens*, *C. suppressalis* and *C. medinalis* are becoming important factors in limiting rice production (Wang, 2012). *S. inferens* and *C. suppressalis* are widely distributed in the Eurasian countries and cause enormous economic losses annually, and *C. medinalis* is widely distributed in most rice growing regions, and mainly cause damage south of the Qinling‐Huaihe Line (Zhang *et a1*., 1980, 1981a,b). In China, brown planthopper, *N. lugens*, and white‐backed planthopper, *S. furcifera*, also cause severe damage in most rice growing regions (18–40°N) (Hu *et al*., 1988; Qi *et al*., 2010). For a long time, control of these insect pests has depended mainly on the use of large amounts of chemical insecticides, mostly as cover sprays, which resulted in heavily environmental pollution and represented a health hazard to farmers as well as significantly increasing the cost of rice production (Tang *et al*., 2006). Consequently, the use of ecologically intensive approaches for controlling insect pests has been given more attention.

Intercropping of rice with other crops, or with complex planting layout, is difficult for mechanical direct‐sowing, thus the mixed seeding of different rice cultivars may be much more efficient and logistically manageable. In this study, potential effects of crop composition shift as a result of increasing acreage of *Bt* rice on target stemborers (*S. inferens* and *C. suppressalis*) and leafrollers (*C. medinalis*), and non‐target planthoppers (*N. lugens* and *S. furcifera*), were assessed at six intra‐specific seed mixture treatments of *Bt* rice (100%, 95%, 90%, 80%, 60%, 0%) with its parental line. We investigated population dynamics of target and non‐target pests and rice grain yield in different seed mixture sowing to find a reasonable mixture in cultivar diversity (i.e., inter‐varietal diversity with transgenic *Bt* rice and its non‐transgenic parental line) for controlling population density of the target and even non‐target agricultural insect pests, simultaneously reducing the agrochemical input. The specific objectives of this study were: (i) to determine the appropriate seed mixture ratio(s) to achieve the optimum suppression of the target stemborers and leafrollers while maintaining the rice grain yield; and (ii) to quantify the effect of intra‐specific genetic diversity on non‐target planthoppers.

## Materials and methods

### Rice cultivars

The transgenic *Bt* rice (cv. Huahui1 with *cry1Ab/cry1Ac* gene, named as Bt) has been known to confer significant resistance to stemborers (*S. inferens*, *C. suppressalis*) and leafrollers (*C. medinalis*) as its target species (Cui & Zhang, 2008; Tian, 2010; Li *et al*., 2012; Guo *et al*., 2013), and the non‐transgenic rice (cv. Minghui63, named as Nt) is the parental line; both rice cultivars were obtained from the College of Plant Science and Technology, Huazhong Agricultural University. Both transgenic *Bt* rice and non‐transgenic rice cultivars used in this study possessed identical growing periods (approximately 137 days) and were well adapted to the growing conditions of southern China.

### Field experiment

**Figure 1:**
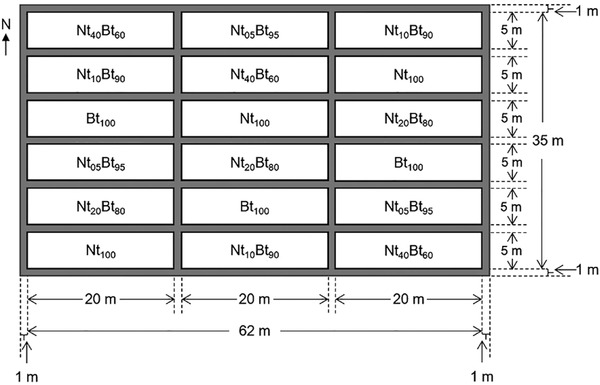
Field layout and deployment of the six seed mixture treatments of transgenic *Bt* rice (cv. Huahui1, with *cry1Ab/cry1Ac* gene, named as Bt) and its parental line of non‐transgenic rice (cv. Minghui63, named as Nt) (Bt_100_–100% *Bt* rice, Nt_05_Bt_95_–5% non‐transgenic and 95% *Bt* rice, Nt_10_Bt_90_–10% non‐transgenic and 90% *Bt* rice, Nt_20_Bt_80_–20% non‐transgenic and 80% *Bt* rice, Nt_40_Bt_60_–40% non‐transgenic and 60% *Bt* rice, Nt_100_–100% non‐transgenic rice; three replications per treatment. The same format is used for the subsequent figures).

This experiment was conducted from 2013 to 2014 in the field station of the Institute of Plant Protection, Chinese Academy of Agricultural Sciences, located at the Maiyuan Village (25°36′2.02′′N, 110°41′45.07′′E), Xinan County, Guangxi Zhuang Autonomous Region of Southern China. Six treatments with different seed mixture ratios of *Bt* (Bt) and non‐transgenic (Nt) rice were evaluated, including 100% *Bt* rice (Bt_100_), 5% Nt and 95% *Bt* (Nt_05_Bt_95_), 10% Nt and 90% *Bt* (Nt_10_Bt_90_), 20% Nt and 80% *Bt* (Nt_20_Bt_80_), 40% Nt and 60% *Bt* (Nt_40_Bt_60_) and 100% Nt rice (Nt_100_), and each treatment was replicated three times. Each experimental field unit (six treatments × three replications = 18 total plots) consisted of a 5 m wide and 20 m long plot with uniform field gradient for homogeneous irrigation. The experimental layout consisted of three passes or three rows of six plots in north‐south direction, each row of six plots representing a replication. The six treatments were randomly assigned to six plots within each replication. Plots were separated by a 1 m ditch between treatment blocks and plots for irrigation and to provide a blank barrier (seen in Fig. 1).

Six seed mixture treatments were sown in six seedling beds (2 m × 6 m) on May 26 in 2013 and 2014 respectively, and then transplanted to the corresponding experimental plots when seedlings were 30 days old. All experimental plots were fertilized with 7.5 g/m^2^ compound fertilizer (N : P : K = 18 : 15 : 12) and 7.5 g/m^2^ urea before rice transplanting and 15 days post‐transplanting, respectively. Prior to rice transplanting, experimental plots were sprayed with 22.5 g/m^2^ pentachlorophenol sodium powder (65%) for controlling ampullariidae. After rice transplanting, no pesticides were applied and the manual weeding once in early tillering stage kept the field weed‐free during the entire growing season of the rice crop.

### Population dynamics of the target and non‐target pests

#### Population dynamics of the target stemborers

Field investigation and sampling were conducted weekly from July 29 to September 30 in both study years. Three sub‐samples were taken from each experimental plot, with a total of 54 samples per sample date (18 experimental plots × three repeated samples per plot). Twenty plants per plot were randomly selected. The sampled rice plants were cut at the base of the plant and collected to count and record the larvae of target stemborers, *S. inferens* and *C. suppressalis*. Based on the characteristic of oviposition and feeding behaviors of *S. inferens* and *C. suppressalis*, that is, female moths oviposit on rice leaves, leaf sheaths and stems, and the eggs hatch and larvae feed on the damaged rice tissues (Jiang *et al*., 2005); the damaged plant tissues of sampled rice plants were dissected by using a sharp scalpel and then the larvae numbers were counted and recorded. Larval densities were calculated as number per 100 plants for each stemborer species.

#### Population dynamics of the target leafrollers

During the investigation of the target stemborers, the larvae of the target leafroller *C. medinalis*, were also counted and recorded simultaneously on the above collected rice plants. As noted for stemborers, *C. medinalis* also showed species‐specific characteristic of oviposition and damage behavior, that is, the female moths oviposit on folded leaves, the eggs hatch on and then larvae fold rice leaves and then feed in the folded leaves (Jiang, 2011; Punithavalli *et al*., 2014). Therefore, the folded leaves of the above collected rice plants were expanded manually and counted, and the larvae number recorded. The density of the *C. medinalis* larvae was also calculated as number per 100 plants.

#### Population dynamics of the non‐target planthoppers

Before the investigation of target stemborers and leafrollers, the above sampled 20 plants per plot were selected to count and record both nymphs and adults of non‐target planthoppers, *N. lugens* and *S. furcifera* by using the plant flapping technique (patting rice plants manually to make planthoppers dropping into a cover screen and white tray) weekly from July 29 to September 30 in 2013 and 2014, respectively. Samples were collected in the field and the specimens were placed back into the same experimental plot from which the samples were collected. Based on the three 20‐plant samples per plot, population abundances of *N. lugens* and *S. furcifera* were calculated as number per 100 plants.

### Rice damage of target stemborers and leafrollers

#### Rice damage of the target stemborers

During the investigation of population dynamics of the target stemborers, the numbers of dead heart and white head tillers, typical damage caused by *S. inferens* and *C. suppressalis*, were counted and recorded to estimate their respective damage. In addition, the total numbers of damaged rice plants with larvae of *S. inferens* and *C. suppressalis* were also respectively counted and recorded to calculate the rate of damaged plants by the target stemborers as follows:

Rate  of  damaged  plants %= Number  of  damaged  plants  Total  number  of  sampled  plants 20×100%,

Rate  of  dead  heart  and  white  head %= Number  of  dead  heart  and  white  head  tillers  Total  number  of  tillers  in 20 plants ×100%.

#### Rice damage of the target leafrollers

During the investigation of population dynamics of the target leafroller, the number of tillers with folded leaves, typical damage symptom of *C. medinalis*, was counted and recorded to estimate the leafroller damage of plants. The rice damage induced by leafrollers was calculated as follows:

Rate  of  damaged  plants  with  folded  leaves %= Number  of  damaged  plants  with  folded  leaves  Total  number  of  sampled  plants 20×100%,

Rate  of  folded  leaves %= Number  of  folded  leaves  in 20 plants  Number  of 20 plant s′ total  leaves ×100%.

### Rice yields

Two indexes were measured to evaluate the rice yield, that is, 1000‐grain dry weight (g; grain plumpness) and grain dry weight per 100 plants (g; economic yield). Test plots were harvested on October 1 each year. Three repeated samples of 100 plants were randomly harvested from each plot. The harvested ears of each sampled plant was dried at 80°C for 72 h to measure 1000‐grain dry weight (six repeats for three plots of each seed mixture treatment) and grain dry weight per 100 plants (three repeats for three plots of each seed mixture treatment) using an automatic electrobalance (Model: BN0100; range: 0–220 g [precision: 0.1 mg] and 0–5 kg [precision: 0.1 g]; Wenzhou Baien Instrument Co., Ltd; Zhejiang Province of China).

### Data analysis

All data were analyzed using the statistical software SPSS 19.0 (2015, SPSS Institute Inc., Chicago, IL, USA). Three‐way repeated‐measure analysis of variance (ANOVA) was used to analyze the effects of seed mixture ratios (Bt_100_, Nt_05_Bt_95_, Nt_10_Bt_90_, Nt_20_Bt_80_, Nt_40_Bt_60_ and Nt_100_), insect species (target stemborer: *S. inferens* vs. *C. suppressalis*; non‐target planthopper: *N. lugens* vs. *S. furcifera*), sampling year (2013 vs. 2014), and their interactions on population dynamics of the stemborers and planthoppers, and on the rice damage indexes (i.e., the rate of dead heart and white head tillers and the rate of damaged plants) of target stemborers. In addition, two‐way repeated‐measure ANOVAs were also used to examine the effects of seed mixture ratios, sampling year, and their interactions on population dynamics and rice damage index (i.e., the rate of folded leaves and the rate of damaged plants with folded leaves) of the target leafrollers. Furthermore, two‐way ANOVAs were also used to study the effects of seed mixture ratios, sampling year and their interactions on 1000‐grain dry weight and grain dry weight per 100 plants. The differences in the population dynamics and rice damage indexes of target stemborers and leafrollers, and population dynamics of the non‐target planthoppers among the six levels of seed mixture ratios, between two species of target stemborers or non‐target planthoppers, and between two sampling years, were separated by the group‐paired *t*‐test at *P *< 0.05. The differences in 1000‐grain dry weight and grain dry weight per 100 plants among the six levels of seed mixture ratios, between two species of target stemborers or non‐target planthoppers, were separated by the *t*‐test at *P *< 0.05. Abundance data were log‐transformed and percent data were arcsine‐transformed to normalize the data prior to analysis.

## Results

### Effects of seed mixture sowing with Bt rice and non‐transgenic rice on population dynamics of the target insect pests

#### Population dynamics of the target stemborers

**Figure 2:**
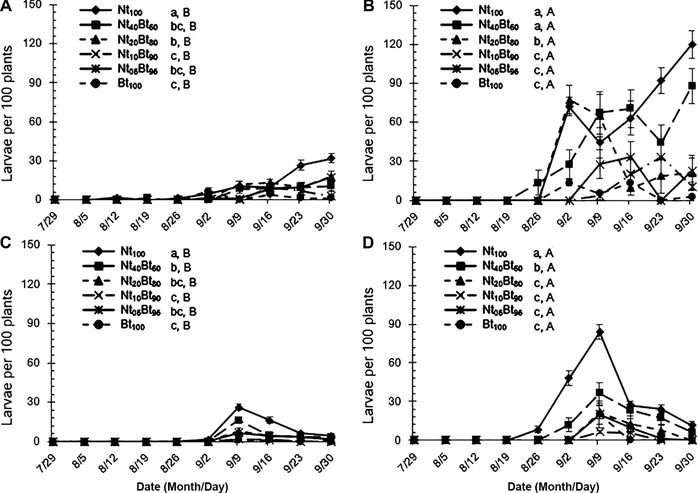
Population dynamics of the target rice stemborers, *Sesamia inferens* (A‐2013; C‐2014) and *Chilo suppressalis* (B‐2013; D‐2014) in the paddyfields as influenced by six ratios of seed mixture sowing with transgenic *Bt* rice (cv. Huahui1 with *cry1Ab/cry1Ac* gene, termed as Bt) and its parental line of non‐transgenic rice (cv. MingHui63; termed as Nt) from July 29 to September 30, 2013 and 2014 (Bt_100_–100% *Bt* rice, Nt_05_Bt_95_–5% non‐transgenic and 95% *Bt* rice, Nt_10_Bt_90_–10% non‐transgenic and 90% *Bt* rice, Nt_20_Bt_80_–20% non‐transgenic and 80% *Bt* rice, Nt_40_Bt_60_–40% non‐transgenic and 60% *Bt* rice, Nt_100_–100% non‐transgenic rice. Different lowercase and uppercase letters indicated significant differences among the treatments of seed mixture sowing for *S. inferens* and *C. suppressalis*, and between *S. inferens* and *C. suppressalis* within seed mixture treatment (group‐paired *t*‐test at *P*<0.05), respectively. The same format is used for the subsequent figures).

Seed mixture ratio, stemborer species, sampling year and their interactions all significantly affected population dynamics of the target stemborers (*P *< 0.001; Table 1). The occurrence of *C. suppressalis* was more serious than that of *S. inferens* in both years (*P *< 0.001; Fig. 2), and the occurrences of both stemborer species were more severe in 2013 than 2014. Population of *S. inferens* began to increase after early September (Fig. 2A and C) while *C. suppressalis* activity occurred about two weeks earlier (Fig. 2B and D); population abundances of both stemborer species decreased with increased ratios of resistant rice in the seed mixture sowing treatments (Fig. 2).

**Table 1:** Three‐way repeated‐measure analysis of variances (ANOVAs) on population dynamics of the target stemborers (*Sesamia inferens* and *Chilo suppressalis*) and non‐target planthoppers (*Nilaparvata lugens* and *Sogatella furcifera*), and on rice damage caused by target stemborers with seed mixture ratios, insect species and sampling years as main factors and their interactions; and two‐way repeated‐measure ANOVAs on population dynamics and damage caused by target leafrollers, *Cnaphalocrocis medinalis* with seed mixture ratio and year as main factors and their interactions; and two‐way ANOVAs on rice yield with seed mixture ratio and year as main factors and their interactions (*F*/*P* values)

Measured indexes		Ratios (R)†	Species (S)‡	Year (Y)§	R × S	R × Y	S × Y	R × S × Y
Population abundance (no. per 100 plants)	Rice stemborers	52.1/<0.001***	163.5/<0.001***	96.6/<0.001***	23.1/<0.001***	7.22/<0.001***	46.2/<0.001***	6.16/<0.001***
	Rice leafrollers	74.6/<0.001***	/	0.35/0.56	/	11.5/<0.001***	/	/
	Rice planthoppers	652.1/<0.001***	8.01/<0.001***	10.6/0.002***	1.90/<0.001***	5.01/0.001***	86.4/<0.001***	2.15/0.005***
Damage rate (%)	The rate of damaged plants by stemborers	22.6/<0.001***	133.3/<0.001***	10.6/0.002***	8.06/<0.001***	3.05/0.018**	73.7/<0.001***	3.52/0.009***
	The rate of dead heart and white head tillers by stemborers	23.2/<0.001***	56.1/<0.001***	1.38/0.25	8.66/<0.001***	0.75/0.59	8.84/0.005***	0.80/0.008***
	The rate of damaged plants with folded leaves by leafrollers	85.6/<0.001***	/	15.5/0.001***	/	21.9/<0.001***	/	/
	The rate of folded leaves by leafrollers	99.0/<0.001***	/	1.50/<0.23	/	37.6/<0.001***	/	/
Rice yield (g)	1000‐grain dry weight	37.0/<0.001***	/	22.0/<0.001***	/	1.14/0.35	/	/
	Grain dry weight per 100 plants	78.6/<0.001***	/	5.43/0.029**	/	0.44/0.82	/	/

^†^Ratios – Six seed mixture ratios of transgenic *Bt* rice and its non‐transgenic parental line (100% *Bt*, 5% non‐transgenic and 95% *Bt*, 10% non‐transgenic and 90% *Bt*, 20% non‐transgenic and 80% *Bt*, 40% non‐transgenic and 60% *Bt*, and 100% non‐transgenic rice).

^‡^Species – Two species of target stemborers, *S. inferens* and *C. suppressalis*, and two species of non‐target planthoppers, *N. lugens* and *S. furcifera*.

^§^Year – 2013 and 2014; ^*^ *P* < 0.10; ^**^ *P* < 0.05; ^***^ *P* < 0.01; / not applicable.

Population abundance of *C. suppressalis* was significantly higher than that of *S. inferens* under the same seed mixture treatment in both years (*P *< 0.05; Fig. 2). Population abundances of *S. inferens* (2013 and 2014; Fig. 2A and C) and *C. suppressalis* (2014; Fig. 2D) were highest in Nt_100_ and significantly higher than those in other seed mixture treatments (*P *< 0.05), whereas population abundances of *S. inferens* and *C. suppressalis* were lowest in Bt_100_ and significantly lower than those in Nt_20_Bt_80_, Nt_40_Bt_60_ and Nt_100_ in 2013 (*P *< 0.05; Fig. 2A and B) and in Nt_40_Bt_60_ and Nt_100_ in 2014 (*P *< 0.05; Fig. 2C and D), respectively. Moreover, there were no significant differences in population abundances of *S. inferens* and *C. suppressalis* in Nt_10_Bt_90_ and Nt_05_Bt_95_ in 2013 (*P *> 0.05; Fig. 2A and B) and Nt_05_Bt_95_, Nt_10_Bt_90_ and Nt_20_Bt_80_ in 2014 (*P *> 0.05; Fig. 2C and D) compared with that in Bt_100_.

#### Population dynamics of the target leafrollers

**Figure 3:**
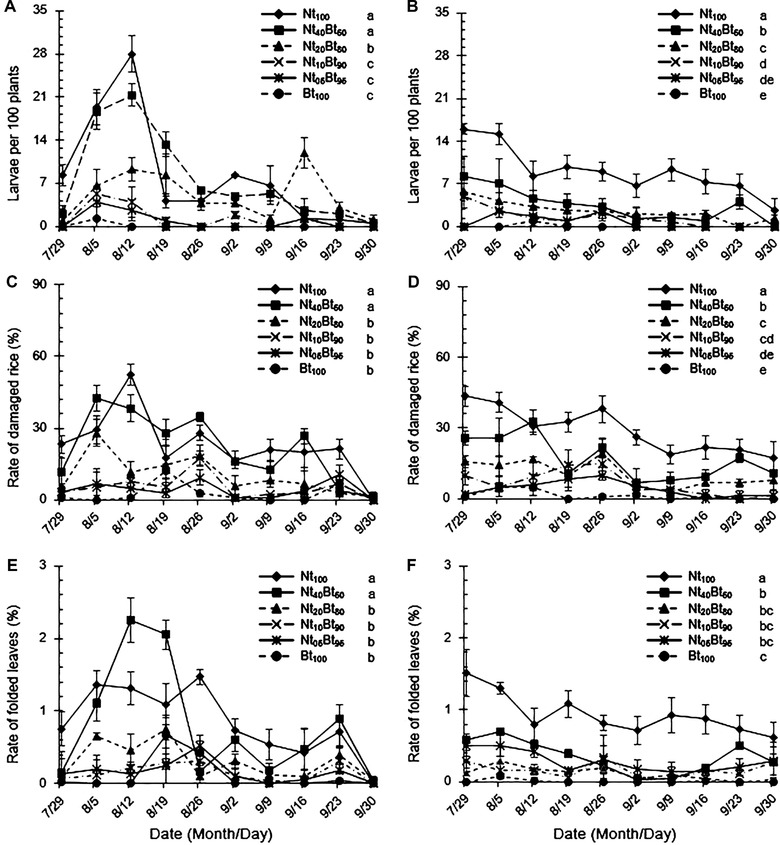
Population dynamics (A and B) of the target rice leafroller, *Cnaphalocrocis medinalis*, the rate of damaged rice plants with folded leaves (C and D) and the rate of folded leaves (E and F) caused by *C. medinalis* in the paddyfields under six ratios of seed mixture sowing with transgenic *Bt* rice and its non‐transgenic parental line from July 29 to September 30 in 2013 (A, C and E) and 2014 (B, D and F). Different lowercase letters indicated significant differences among the seed mixture sowing treatments for each leafroller species using group‐paired *t*‐test at *P*<0.05. Subsequent figures follow the same format.

Significant effects (*P *< 0.001) of seed mixture ratio and its interactions with sampling year on the population dynamics of *C. medinalis* were observed and are shown in Table 1. Population abundance of *C. medinalis* did not significantly differ between 2013 and 2014 (*P* = 0.56 > 0.10; Fig. 3A and B) and the larval abundance of *C. medinalis* declined after August 12 in 2013 and July 29 in 2014 (Fig. 3A and B). Population abundance of *C. medinalis* was lowest in Bt_100_ and significantly lower than those in Nt_20_Bt_80_‐Nt_100_ in 2013 (*P* < 0.05; Fig. 3A) and Nt_10_Bt_90_‐Nt_100_ in 2014 (*P* < 0.05; Fig. 3B). Moreover, there were no significant differences in population abundances of *C. medinalis* in Nt_05_Bt_95_ and Nt_10_Bt_90_ in 2013 (*P* > 0.05; Fig. 3A) and Nt_05_Bt_95_ in 2014 (*P* > 0.05; Fig. 3B).

### Effects of seed mixture sowing with Bt rice and non‐transgenic rice on population dynamics of the non‐target planthoppers

**Figure 4:**
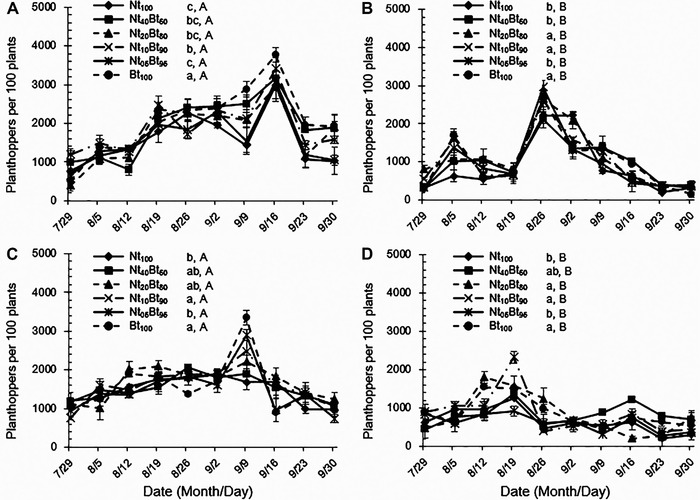
Population abundances of non‐target rice planthoppers, *Nilaparvata lugens* (A‐2013; C‐2014) and *Sogatella furcifera* (B‐2013; D‐2014), in the paddyfields under six ratios of seed mixture sowing with transgenic *Bt* rice and its non‐transgenic parental line from July 29 to September 30, 2013 and 2014.

There were significant effects of seed mixture ratio, planthopper species, sampling year and their interactions on the population dynamics of non‐target planthoppers (*P* < 0.01; Table 1). The occurrences of these two planthopper species were greater in 2013 compared to those in 2014, and the population abundance of *N. lugens* was significantly higher than that of *S. furcifera* for the same seed mixture treatment in both years (*P* < 0.05; Fig. 4). Population abundances of both planthopper species were lowest in Nt_05_Bt_95_ and significantly lower than those in Nt_10_T_90_ and Bt_100_ in both years (*P* < 0.05; Fig. 4A–D). Moreover, there were no significant differences in population abundances of *N. lugens* and *S. furcifera* between Nt_05_Bt_95_ and Nt_100_, respectively (*P* > 0.05; Fig. 4A–D).

### Effects of seed mixture sowing with Bt rice and non‐transgenic rice on plant damage by target stemborers

#### The rate of damaged plants

**Figure 5:**
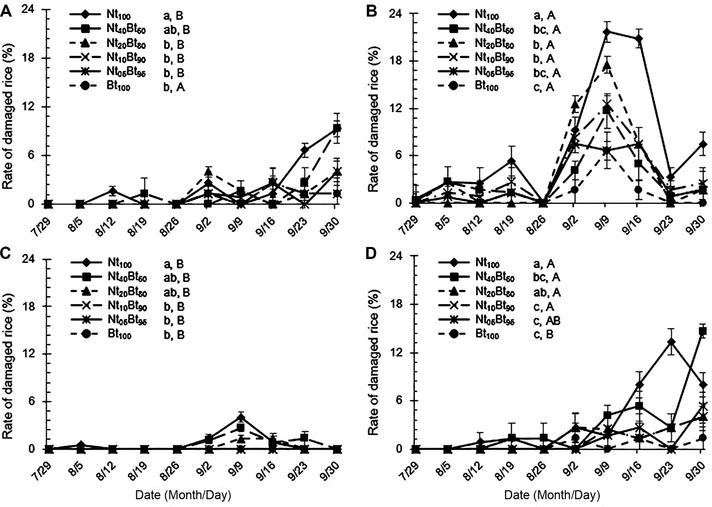
The rate of plant damage caused by the target rice stemborers, *Sesamia inferens* (A‐2013; C‐2014) and *Chilo suppressalis* (B‐2013; D‐2014), in paddyfields under six ratios of seed mixture treatments with transgenic *Bt* rice and its non‐transgenic parental line from July 29 to September 30, 2013 and 2014.

Damage inflicted by rice stemborers significantly varied with seed mixture ratios, stemborer species, sampling year and their interactions (*P* < 0.05 or *P* < 0.01; Table 1). The rate of damaged plants increased after August 26 in 2013 and September 2 in 2014, and was significantly higher in 2013 than that in 2014 for both stemborer species (*P* < 0.05; Fig. 5). The rate of damaged plants by *C. suppressalis* was significantly higher than that by *S. inferens* under the same seed mixture sowing treatment, except for Nt_05_Bt_95_ and Bt_100_ in 2014 (*P* < 0.05; Fig. 5). The rate of damaged plants by *S. inferens* was the highest in Nt_100_ and significantly higher than those in other seed mixture sowing treatments (*P* < 0.05), except for Nt_40_Bt_60_ in 2013 (*P* > 0.05; Fig. 5A) and in Nt_20_Bt_80_ and Nt_40_Bt_60_ in 2014 (*P* > 0.05; Fig. 5C). The rate of damaged plants by *C. suppressalis* was the highest in Nt_100_ and significantly higher than those in other seed mixture treatments (*P* < 0.05), except for Nt_20_Bt_80_ in 2014 (*P* > 0.05; Fig. 5D). Moreover, the rate of damaged plants by *S. inferens* was the lowest in Bt_100_ and there was no significant difference between Bt_100_ and Nt_05_Bt_95_, Nt_10_Bt_90_ or Nt_20_Bt_80_ in 2013 (*P* > 0.05; Fig. 5A), and between Bt_100_ and Nt_05_Bt_95_ or Nt_10_Bt_90_ in 2014 (*P* > 0.05; Fig. 5C). The rate of damaged plants by *C. suppressalis* was the lowest in Bt_100_ in 2013 (Fig. 5B) and significantly lower than those in other seed mixture treatments except for Nt_05_Bt_95_ and Nt_10_Bt_90_ in 2014 (*P* < 0.05; Fig. 5D).

#### The rate of dead heart and white head tillers

**Figure 6:**
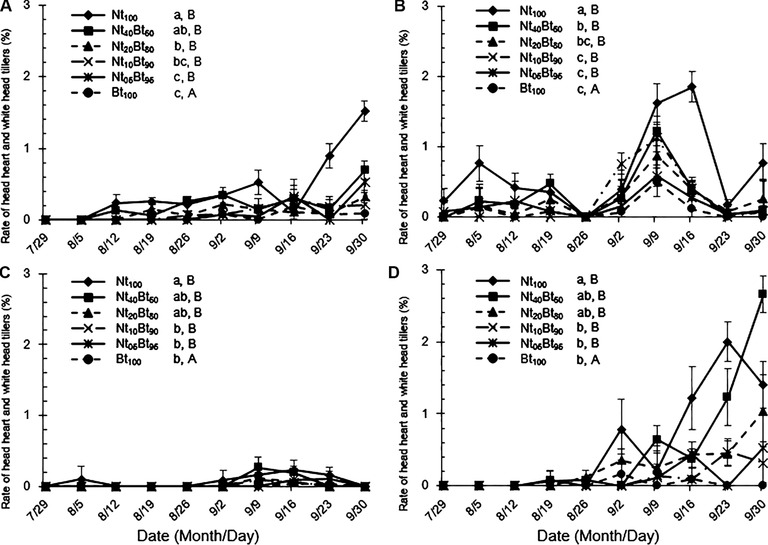
The rate of dead heart and white head tillers caused by rice stemborers, *Sesamia inferens* (A‐2013; C‐2014) and *Chilo suppressalis* (B‐2013; D‐2014), in the paddyfields under six ratios of seed mixture treatments with transgenic *Bt* rice and its non‐transgenic parental line from July 29 to September 30, 2013 and 2014.

The rate of stemborer‐induced dead heart and white head tillers was significantly influenced by seed mixture ratios and stemborer species (*P* < 0.001), and the interaction of seed mixture, stemborer species and sampling year (*P* < 0.01), but not the sampling year (*P* = 0.25 > 0.05) and the interaction between seed mixture ratios and sampling year (*P* = 0.59 > 0.05) (Table 1). The rate of dead heart and white head tillers by these two stemborer species increased conspicuously after August 5 in 2013, whereas the damage severity was delayed by 3 weeks in 2014 (Fig. 6). The rate of dead heart and white head tillers by *C. suppressalis* was significantly higher than that by *S. inferens* under the same seed mixture treatment for both years (*P* < 0.05; Fig. 6). The rate of dead heart and white head tillers was the highest in Nt_100_ and the lowest in Bt_100_ and Nt_05_Bt_95_ for both species in both years (Fig. 6A–D), while there was no significant difference between Bt_100_ and Nt_05_Bt_95_ in 2013 (*P* > 0.05; Fig. 6A and B) and between Bt_100_ and Nt_05_Bt_95_ or Nt_10_Bt_90_ in 2014 (*P* > 0.05; Fig. 6C and D).

### Effects of seed mixture sowing with Bt rice and non‐transgenic rice on plant damage caused by leafrollers

#### The rate of damaged rice plants with folded leaves

Seed mixture ratios and their interaction with sampling year significantly affected the rate of damaged plants with folded leaves (*P* < 0.001; Table 1). The rate of damage caused by *C. medinalis* was the highest in Nt_100_, and significantly higher than those in other seed mixture ratios (*P* < 0.05), except for Nt_40_Bt_60_ in 2013 (*P* > 0.05; Fig. 3C). The damage rate was the lowest in Bt_100_, and there was no significant difference between Bt_100_ and Nt_05_Bt_95_, Nt_10_Bt_90_ or Nt_20_Bt_80_ in 2013 (*P* > 0.05; Fig. 3C) and between Bt_100_ and Nt_05_Bt_95_ in 2014 (*P* > 0.05; Fig. 3D).

#### The rate of folded leaves

There were significant effects of seed mixture ratios and their interaction with sampling year on the rate of folded leaves caused by *C. medinalis* (*P* < 0.001; Table 1). The rate of folded leaves was the highest in Nt_100_, and significantly higher than those in other seed mixture sowing treatments (*P* < 0.05) in both years, except for Nt_40_Bt_60_ in 2013 (*P* > 0.05; Fig. 3E). The rate of folded leaves was the lowest in Bt_100_, and there was no significant difference between Bt_100_ and Nt_05_Bt_95_, Nt_10_Bt_90_ or Nt_20_Bt_80_ for both years (*P* > 0.05; Fig. 3E and F).

### Effects of different seed mixture ratios with Bt rice and non‐transgenic rice on yield

#### 1000‐grain dry weight

**Figure 7:**
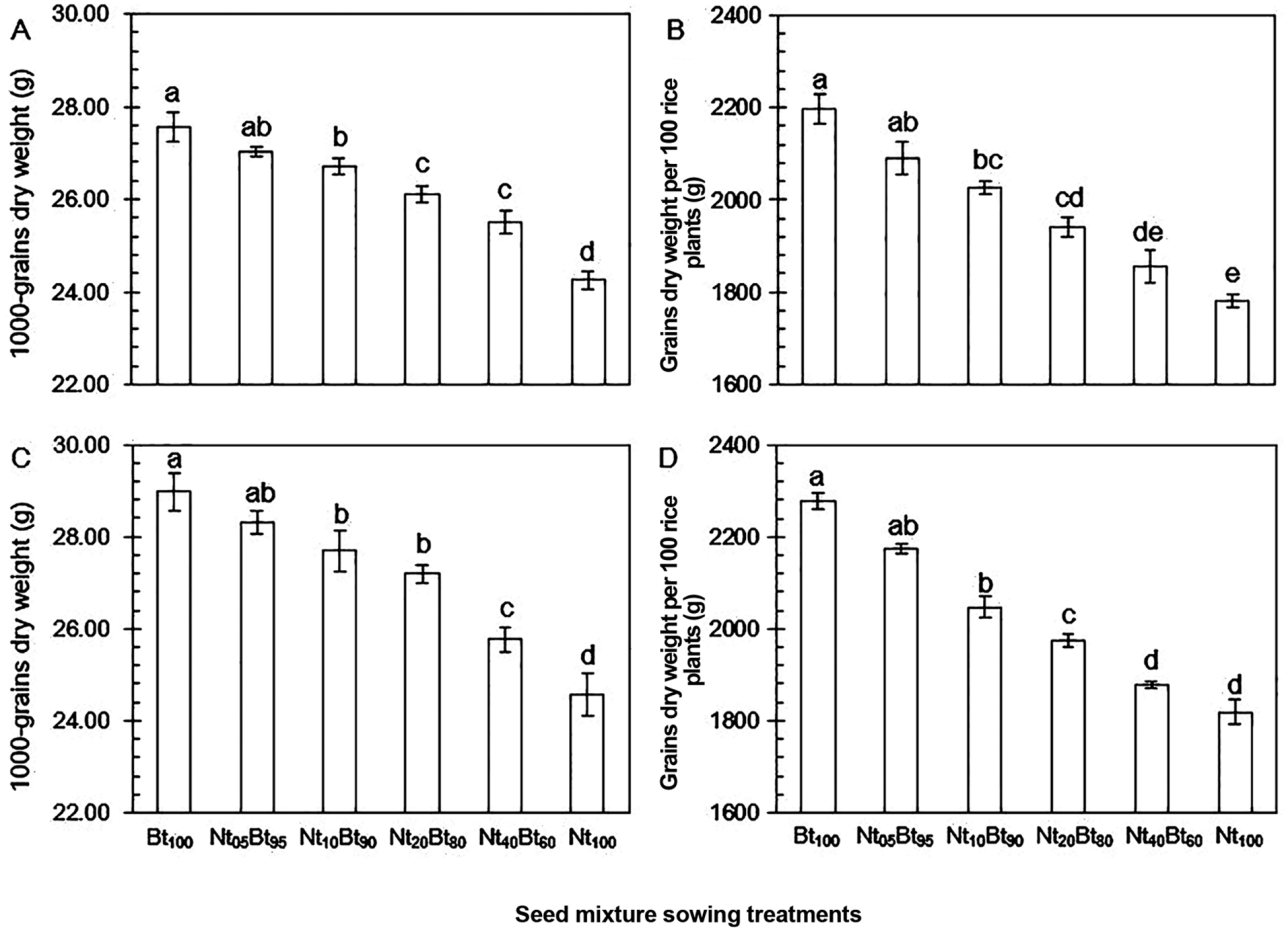
Rice grain yield, measured in terms of 1000‐grain dry weight (A and C) and grain dry weight per 100 rice plants (B and D), under six seed mixture ratios with transgenic *Bt* rice and its non‐transgenic parental line in 2013 (A and B) and 2014 (C and D). Different lowercase letters indicated significant differences in grain yield among seed mixture treatments (Duncan multiple range test, *P*<0.05).

Significant effects of seed mixture ratios and sampling year on 1000‐grain dry weight were observed (*P* < 0.001; Table 1). The 1000‐grain dry weight was the highest in Bt_100_ (2013: 27.57 g; 2014: 28.98 g) and the lowest in Nt_100_ (2013: 24.27 g; 2014: 24.56 g) (Fig. 7A and C), and there was no significant difference between Bt_100_ and Nt_05_Bt_95_ (2013: 27.02 g; 2014: 28.31 g) (*P* > 0.05; Fig. 7A and C). The 1000‐grain dry weight was higher in 2014 than that in 2013 for all six seed mixture treatments (Fig. 7A and C).

#### Grain dry weight per 100 plants

Seed mixture ratios (*P* < 0.001) and sampling year (*P* < 0.05) significantly affected grain dry weight per 100 plants (Table 1). Grain dry weight per 100 plants was the highest in Bt_100_ (2013: 2197.1 g; 2014: 2276.5 g) and the lowest in Nt_100_ (2013: 1780.8 g; 2014: 1818.2 g) (Fig. 7B and D), and there was no significant difference between Bt_100_ and Nt_05_Bt_95_ (2013: 2096.6 g; 2014: 2175.5 g) (*P* > 0.05; Fig. 7B and D). Overall, the grain dry weight per 100 plants was lower in 2013 than that in 2014 for all six seed mixture treatments (Fig. 7B and D).

### Correlation between rice yield and population abundances of target and non‐target insect pests and their damage parameters

Pearson's correlation analysis indicated that there were significant negative correlations between the population abundances of target pests (stemborers and leafrollers) and rice yield indexes of 1000‐grain dry weight (*S. inferens*: *r* = −0.90, *P* = 0.014 < 0.05; *C. suppressalis*: *r* = −0.98, *P* = 0.0012 < 0.01; *C. medinalis*: *r* = −0.96, *P* = 0.002 < 0.01) and grain dry weight per 100 plants (*S. inferens*: *r* = −0.84, *P* = 0.036 < 0.05; *C. suppressalis*: *r* = −0.91, *P* = 0.012 < 0.05; *C. medinalis*: *r* = −0.87, *P* = 0.025 < 0.05) (Table 2). However, there were no significant negative correlations between the population abundances of non‐target planthoppers and rice yield indexes of 1000‐grain dry weight (*N. lugens*: *r* = −0.50, *P* = 0.31 > 0.10; *S. furcifera*: *r* = −0.19, *P* = 0.71 > 0.10) and grain dry weight per 100 plants (*N. lugens*: *r* = −0.51, *P* = 0.31 > 0.10; *S. furcifera*: *r* = −0.35, *P* = 0.49 > 0.10) (Table 2).

**Table 2:** Pearson's correlation between rice grain yield (1000‐grain dry weight and grain dry weight per 100 rice plants) and population abundances of the target stemborers (*Sesamia inferens* and *Chilo suppressalis*) and leafrollers (*Cnaphalocrocis medinalis*), non‐target planthoppers (*Nilaparvata lugens* and *Sogatella furcifera*), and between rice yield and insect‐induced damage parameters (*r*/*P* values)

Measured indexes		1000‐grain dry weight (g)	Grain dry weight per 100 plants (g)
Population abundance (no. per 100 plants)	Stemborer, *S. inferens*	−0.90/0.014**	−0.84/0.036**
	Stemborer, *C. suppressalis*	−0.98/0.001***	−0.91/0.012**
	Leafroller, *C. medinalis*	−0.96/0.002***	−0.87/0.025**
	Planthopper, *N. lugens*	−0.50/0.31	−0.51/0.31
	Planthopper, *S. furcifera*	−0.19/0.71	−0.35/0.49
Damage rate (%)	The rate of damaged plants by *S. inferens*	−0.73/0.097*	−0.83/0.043**
	The rate of damaged plants by *C. suppressalis*	−0.71/0.018**	−0.76/0.078*
	The rate of damaged plants with folded leaves by *C. medinalis*	−0.96/0.003***	−0.85/0.033**
	The rate of dead heart and white head tillers by *S. inferens*	−0.82/0.045**	−0.70/0.077*
	The rate of dead heart and white head tillers by *C. suppressalis*	−0.90/0.015**	−0.79/0.062*
	The rate of folded leaves by *C. medinalis*	−0.88/0.021**	−0.75/0.086*

^*^ *P* < 0.10; ^**^ *P* < 0.05; ^***^ *P* < 0.01.

The rate of damaged plants by target stemborers negatively correlated with 1000‐grain dry weight (*S. inferens*: *r* = −0.73, *P* = 0.097 < 0.10; *C. suppressalis*: *r* = −0.71, *P* = 0.018 < 0.05) as well with grain dry weight per 100 plants (*S. inferens*: *r* = −0.83, *P* = 0.043 < 0.05; *C. suppressalis*: *r* = −0.76, *P* = 0.078 < 0.10). Moreover, the rate of dead heart and white head tillers negatively correlated with the 1000‐grain dry weight (*S. inferens*: *r* = −0.82, *P* = 0.045 < 0.05; *C. suppressalis*: *r* = −0.90, *P* = 0.015 < 0.05) as well with grain dry weight per 100 plants (*S. inferens*: *r* = −0.70, *P* = 0.077 < 0.10; *C. suppressalis*: *r* = −0.79, *P* = 0.062 < 0.10). In addition, both rice yield indexes negatively correlated with the rate of damaged plants (1000‐grain dry weight: *r* = −0.96, *P* = 0.003 < 0.01; grain dry weight per 100 plants: *r* = −0.85, *P* = 0.033 < 0.05) and the rate of folded leaves (1000‐grain dry weight: *r* = −0.88, *P* = 0.021 < 0.05; grain dry weight per 100 plants: *r* = −0.75, *P* = 0.086 < 0.10) caused by target *C. medinalis* (Table 2).

## Discussion

In China as well as throughout the world, the conversion of semi‐natural habitats to arable land has led to landscape simplification and decreased species diversity, richness, and abundance of natural enemies within agro‐ecosystems (Rand & Tscharntke, 2006; Tscharntke *et al*., 2012). Consequently, landscape simplification has led to a dramatic increase in agricultural insect pest outbreaks, biodiversity loss, and degradation of multiple ecosystem services within agricultural landscapes (Chaplin‐Kramer & Kremen, 2012; Zhao *et al*., 2013). In the past decades, the intensification of agriculture (e.g., cropland expansion) and fragmentation of semi‐natural habitats have become important drivers of biodiversity loss, which have been attributed, at least partially, to pest outbreaks in many agricultural systems (Meehan *et al*., 2011). Numerous studies have demonstrated the potential of using landscape ecology (habitat diversity and complexity) combined with resistant rice cultivars in managing rice insect pests in various agro‐ecosystems (Skovgard & Pats, 1996; Landis *et al*., 2000; Smith & McSorley, 2000). The use of a reasonable mixture to create within‐species genetic diversity is a simple and practicable approach to enhance habitat diversity in ephemeral agricultural production systems (Rand *et al*., 2006; Meehan *et al*. 2011). In this study, transgenic *Bt* rice offered the potential to generate economic benefits for controlling its target insect pests (i.e., stemborers and leafrollers), simultaneously increasing yield.

Many genetically modified rice varieties have been produced (Huang *et al*., 2002; Jia & Peng, 2002) and released into the environment for field‐testing (Messeguer *et al*., 2001, 2004; Chen *et al*., 2004). It is apparent that, as an important crop, transgenic rice varieties will be released for commercial production on a large scale, undoubtedly in the near future (Chen *et al*., 2004; Jia, 2004). In China, genetically modified rice varieties with insect resistance (e.g., *Bt* and *CpTI* genes), disease (*Xa21*) resistance, and herbicide tolerance (*bar*, *EPSPs*) have been developed, and these products are now in the pipeline for commercialization pending approval by the biosafety regulatory agency (Jia, 2002). With the widespread use of transgenic *Bt* rice cultivars, it is also critical to adopt approaches to enhance biodiversity in the field for resistance management as well as to manage secondary pest outbreaks. Therefore, we focused on an examination of an ecological approach (habitat diversity through cultivar mixture) toward enhancing vegetation diversity and its associated functional biodiversity in transgenic rice production systems, which could potentially narrow the gap between sustainable agriculture and biodiversity conservation (Tscharntke *et al*., 2012).

Reasonable mixtures of different crop cultivars can alleviate the limitations triggered by monoculture and low biodiversity (Van & Harfington, 2007) and enhance the natural enemy species richness (Shi *et al*., 2014). It has been suggested that the increase in crop diversity in agricultural landscape configuration contributes to a general decrease in pest damage on crops (Chaplin‐Kramer *et al*., 2011; Gagic *et al*., 2011); the effect may range from localized reduction of pest abundance to a lower general equilibrium of pest populations at the landscape level (Jonsson *et al*., 2012). Because pest population reactions to habitat diversity vary with species complex, mixtures of resistant and susceptible rice cultivars can have different effects on different insect pests (Sheng *et al*., 2016). Simultaneously, the use of seed mixtures has become a common strategy to provide IRM for transgenic *Bt* corn (Burkness *et al*., 2015). Seed mixtures of transgenic and non‐transgenic crops are recommended as a strategy to minimize or eliminate insects’ abilities to develop resistance to insect‐resistant transgenic crops (Ramachandran *et al*., 2000). In this study, we used the seed mixture with transgenic *Bt* rice and its non‐transgenic parental line to sustain the maximum achievable suppression as well as to provide sufficient refuge for the target stemborers and leafrollers to delay their resistance to transgenic rice. As expected, a higher ratio of transgenic *Bt* cutivar in the seed mixture provided better control efficacy for its target stemborers, *S. inferens* and *C. suppressalis*. Population abundances of *S. inferens* and *C. suppressalis* were the lowest in Bt_100_ (100% *Bt* rice), significantly lower than those in the seed mixture treatments with higher ratios of non‐transgenic rice (≥ 20%, including Nt_20_Bt_80_, Nt_40_Bt_60_ and Nt_100_ in 2013; ≥ 40%, including Nt_40_Bt_60_ and Nt_100_ in 2014), while there was no significant difference between Bt_100_ and those treatments with lower ratios of non‐transgenic rice (≤ 10%, including Nt_10_Bt_90_ and Nt_05_Bt_95_ in 2013; ≤ 20%, including Nt_20_Bt_80_, Nt_10_Bt_90_ and Nt_05_Bt_95_ in 2014). The population abundance of the target leafroller, *C. medinalis* was the lowest in Bt_100_ and no significant differences were observed between Bt_100_ and those treatments with lower ratios of non‐transgenic rice (≤ 10%, including Nt_10_Bt_90_ and Nt_05_Bt_95_ in 2013; ≤ 5%, including Nt_05_Bt_95_ in 2014). In this study, the overall occurrence of damage symptoms caused by the target stemborers and leafrollers were consistent with the severity of their larval abundances. Based on this 2‐year study, we demonstrated that the seed mixture sowing with low ratios (≤ 10% or 5%) of non‐transgenic rice provided the same control efficacy as 100% transgenic *Bt* rice (Bt_100_) for the target insect pests, with sufficient level of refuge provided for the resistance management in transgenic rice fields (Onstad *et al*., 2011). Other researchers have also shown similar conclusions wherein the strategy places non‐*Bt* refuge seeds in the bag with transgenic *Bt* rice seeds, typically at a ratio of 5% : 95% (non‐*Bt* : *Bt*) to overcome potential compliance issues that may exist with the use of block or structured refuge (Burkness *et al*., 2015).

The occurrences of *S. inferens* and *C. suppressalis* were the highest in Nt_100_ and the lowest in Bt_100_, and there was no significant difference in planthopper densities between Bt_100_ and Nt_05_Bt_95_. The seed mixture treatment with 95% *Bt* rice and 5% non‐transgenic rice not only had good control efficacy for target stemborers and leafrollers, but also controlled non‐target planthoppers. Monoculture practices have been reported to decrease the abundance of insect's natural enemies and neutral effect on secondary pests (Altieri & Letourneau, 1982), which may result in increased insect pest severity in such systems (Andow, 1991; Landis *et al*., 2000). The seed mixture ratio of Nt_05_Bt_95_ was of great significance for providing a proper refuge for managing the resistance of target pests while controlling primary and secondary pests, simultaneously. In addition, the present study showed that *C. medinalis* was the main rice target pest in August, while *S. inferens* and *C. suppressalis* mainly occurred after September, which corroborates with previous reports from this region (Yang, 2008; Jiang, 2012). For non‐target pests, the population abundance of *N. lugens* captured during the entire sampling period was significantly higher than that of *S. furcifera*, suggesting that the primary economic insect pest of rice in Guangxi is *N. lugens*, which corroborates previous reports from this region (Yang, 2008; Jiang, 2012; Wang *et al*., 2014). The abundances of target and non‐target pests during the entire sampling period in 2013 were higher than those in 2014, which might be attributed to differences in precipitation and wind between the 2 years. Data from China Meteorological Data Sharing Service System (data.cma.cn) showed that heavy thunderstorm events south of the Guangxi region during early October in 2013, and severe thunderstorm events and high wind speed contributed to the wind‐mediated immigration of migratory insects such as *C. medinalis*, *N. lugens* and *S. furcifera* from south China to the Guangxi Zhuang Autonomous Region.

Increasing crop yield and simultaneously optimizing economic profitability are the most important goals of plant science research (Li *et al*., 2011). The integration of legumes into rice‐based cropping systems offers opportunities to increase habitat diversity and sustain productivity and income of smallholder farmers in Southeast Asia (Whitmore *et al*., 2000; Wijnhoud *et al*., 2003). Some studies have suggested that plant genetic diversity provides significant protection from disease in both natural and agricultural ecosystems and may also contribute to increased yield and yield stability in the absence of disease (Mundt & Browning, 1985; Mundt, 1994). For integrated pest management (IPM) in paddyfields, some studies have used mixtures of transgenic and non‐transgenic crop seeds to provide an in‐field refuge for susceptible insects and increase crop yields (Gravois & Helms, 1992; Raboin *et al*., 2012; Tooker & Frank, 2012). By providing a broader base of stress tolerance, varietal diversity may also reduce yield variability when pest infestations or unfavorable weather events occur (Widawsky & Rozelle, 2000). Pearson's correlation analysis indicated that there were significant negative correlations between rice yields and population abundances of target stemborers (*S. inferens* and *C. suppressalis*) and leafrollers (*C. medinalis*) and no significant negative correlations between rice yields and population abundances of non‐target planthoppers (*N. lugens* and *S. furcifera*). In our study, there were lowest population abundances of planthoppers in Nt_100_ and no significant difference between Nt_100_ and Nt_05_Bt_95_; there were lowest population abundances of target pests in Bt_100_ and no significant difference among Bt_100_, Nt_10_Bt_90_ and Nt_05_Bt_95_. We hypothesize that the low density of target pests may provide the ecological niche in favor of the non‐target pests’ occurrence and increased crop yield, and Nt_05_Bt_95_ has good control for both target and non‐target pests. Other researches have shown the similar conclusions (Yu *et al*., 2011; Pan *et al*., 2012; Wang *et al*., 2014). In this study, the 1000‐grain dry weight and grain dry weight per 100 plants were the highest in Bt_100_ and the lowest in Nt_100_ in both years, and there were no significant differences in these yield indexes for seed mixtures with 5% non‐transgenic and 95% *Bt* rice seeds (i.e., Nt_05_Bt_95_) compared with 100% *Bt* rice seed (i.e., Bt_100_). Thus, seed mixture with low ratios (especially 5%) of non‐transgenic rice is advantageous for rice yield stability. Moreover, the 1000‐grain dry weight and grain dry weight per 100 plants in 2014 were both higher than those in 2013 owing to the lower occurrences of the target stemborers (*S. inferens* and *C. suppressalis*) and leafroller *C. medinalis*, and non‐target planthoppers (*N. lugens* and *S. furcifera*) in 2014 compared to 2013.

In summary, the integration of economic and environmental (ecological) parameters is one of the most important characteristics of habitat management, resulting in multiple ecosystem services. A single insect pest‐resistant crop cultivar (e.g., transgenic *Bt* crops) may effectively manage the target pest in a monoculture production system for a short term, but some secondary insect pests may soon become new primary insect pests, emphasizing the need for habitat diversity for sustainability (Ahuja *et al*., 2010). Sustainable agriculture currently faces a challenge from global environmental changes, and the solution to this challenge requires joint forces from farmer associations and landowners (Ostman *et al*., 2001) in addition to academic researchers and industry partnerships. In this study, we found that the use of reasonable mixture in cultivar diversity (i.e., inter‐varietal diversity with transgenic *Bt* rice and its non‐transgenic parental line) is a simple and practicable approach to control population density of the target and even non‐target agricultural insect pests, simultaneously reducing the agrochemical input. In addition, the seed mixture sowing is suggested to provide a proper refuge for susceptible pests, which can reduce the resistance of target pests and be beneficial to the application of *Bt* rice, which remains to be clarified in the future. The adoption of such a simple method by farmers may prove highly valuable in safeguarding the technology. It is also of significance for seed companies to consider supplying seed mixture of transgenic *Bt* rice with ≤ 10% (especially 5%) non‐transgenic parent line of the resistant rice cultivar for famers’ uses (Chi *et al*., 2008).

## Disclosure

The authors declare that they have no conflicts of interest.
